# Prediction of higher mortality reduction for the UK Breast Screening Frequency Trial: a model-based approach on screening intervals

**DOI:** 10.1038/bjc.2011.300

**Published:** 2011-08-23

**Authors:** N T van Ravesteyn, E A M Heijnsdijk, G Draisma, H J de Koning

**Affiliations:** 1 Department of Public Health, Erasmus MC, PO Box 2040, Rotterdam 3000 CA, The Netherlands

**Keywords:** breast cancer, mammography, screening interval, mortality, UK

## Abstract

**Background:**

The optimal interval between two consecutive mammograms is uncertain. The UK Frequency Trial did not show a significant difference in breast cancer mortality between screening every year (study group) and screening every 3 years (control group). In this study, the trial is simulated in order to gain insight into the results of the trial and to predict the effect of different screening intervals on breast cancer mortality.

**Methods:**

UK incidence, life tables and information from the trial were used in the microsimulation model MISCAN–Fadia to simulate the trial and predict the number of breast cancer deaths in each group. To be able to replicate the trial, a relatively low sensitivity had to be assumed.

**Results:**

The model simulated a larger difference in tumour size distribution between the two groups than observed and a relative risk (RR) of 0.83 of dying from breast cancer in the study group compared with the control group. The predicted RR is lower than that reported from the trial (RR 0.93), but within its 95% confidence interval (0.63–1.37).

**Conclusion:**

The present study suggests that there is benefit of shortening the screening interval, although the benefit is probably not large enough to start annual screening.

In randomised controlled trials, mammography screening has been shown to reduce breast cancer mortality rates (Tabar *et al*, 1992; Nystrom *et al*, 1993, 2002). The more frequently a woman has screening exams, the larger the probability of having an early diagnosis, and the larger the mortality reduction might be. However, with more frequent exams, the potential of false-positive exams and overdiagnosis will also increase (Jansen and Zoetelief, 1997; Christiansen *et al*, 2000). There is no consensus on the optimal screening interval (i.e., the time between two consecutive mammograms), as is illustrated by the variety of screening intervals used throughout the world. Most European screening programmes use an interval of 2 years (e.g., The Netherlands, Sweden), whereas other countries use a 3-year interval (United Kingdom, Malta). Even within the same country, screening recommendations vary: in the United States, the American Cancer Society recommends annual screening starting at the age of 40 years (Smith *et al*, 2010), whereas the US Preventive Services Task Force recently changed their recommendation to biennial screening from age 50 to 74 years (US Preventive Services Task Force, 2009).

Two randomised trials compared a 1-year screening interval with a 3-year screening interval, one in women between age 40 and 49 years (Klemi *et al*, 1997) and one in women between age 50 and 62 years (Breast Screening Frequency Trial Group, 2002). The latter, the UK Breast Screening Frequency Trial, was conducted from 1989 to 1996, in order to evaluate the difference in (predicted) breast cancer mortality between screening annually and screening once every 3 years (Breast Screening Frequency Trial Group, 2002). The tumours in the trial group, offered annual screening, were significantly smaller than those diagnosed in the control group, offered screening every 3 years. For node status and histological grade, no significant difference between the two groups was found. The initially reported relative risk (RR) predicted on the basis of two prognostic indices showed a nonsignificant reduction in predicted breast cancer mortality (Breast Screening Frequency Trial Group, 2002). The results were later updated with results on the actual observed number of breast cancer deaths in both groups again showing a nonsignificant reduction in breast cancer mortality. Women in the study group had an RR of 0.93 (95% confidence interval (CI) 0.63–1.37) of dying from breast cancer compared with women in the control group (Duffy and Blamey, 2008).

This finding was (slightly) surprising and raised the question why no significant difference was found between the two groups. It might be that there is truly only a very small mortality benefit of more frequent screening, or there might be other reasons why no difference is found between the two groups, for example, a lack of power or low sensitivity of mammography. Most policy predictions are based on the assumption that increasing the screening frequency will lead to more early diagnoses and consequently in a reduction in breast cancer mortality, hence it is crucial to get more insight in the results of this trial. A simulation model is ideally suited to evaluate the effect of different screening intervals on mortality, because the effect of different screening test sensitivities can be assessed and the model guarantees that trial populations are identical, except for the factors investigated.

In the present study, the UK Breast Screening Frequency Trial was simulated using the microsimulation model MIcrosimulation of SCreening ANalysis–Fatal diameter (MISCAN–Fadia), in order to gain insight into the results of the trial and estimate the effect of different screening intervals on breast cancer mortality.

## Materials and methods

### Model overview

MISCAN–Fadia is a microsimulation model developed within the Cancer Intervention and Surveillance Modeling Network (CISNET) (Tan *et al*, 2006). Briefly, the model simulates life histories in the absence of screening and then assesses how these life histories change as a consequence of screening programmes. MISCAN–Fadia explicitly models invasive tumour growth in combination with the concept of a fatal diameter. The model has been described in detail elsewhere (Tan *et al*, 2006) and information about the model can be found on the CISNET website (http://cisnet.cancer.gov/). A detailed description of the model components and model quantification for the present study is presented in the Appendix.

In brief, for the present study, the model simulates a population of women between the ages of 50 and 62 years in the year 1992 (i.e., the middle year of the trial) using the life tables of the UK female population. Among those who develop breast cancer, the natural history is modelled as a continuously growing tumour. Each tumour has a size (the fatal diameter, which differs between tumours) at which diagnosis and treatment will no longer result in cure given available treatment options. If the tumour is diagnosed (either on the basis of clinical presentation with symptoms or by screening) and treated before it has reached its fatal diameter, the woman will be cured and will die of non-breast cancer causes. Variation between tumours is modelled by probability distributions of tumour growth, threshold diameter of screen detection, clinical diagnosis diameter and fatal disease diameter.

When a screening programme is applied, the pre-clinical tumour may be detected by screening. Each simulated tumour has a diameter at which it will be clinically diagnosed as well as a screen-detection threshold diameter. For the latter, screening test sensitivity is 0% below and 100% above this diameter. The threshold diameter is assumed to decrease with age and calendar year. Screening benefits result from detection of more tumours at a non-fatal size (Tan *et al*, 2006).

### Model calibration and validation

Several approaches have been used to assess the internal reliability of MISCAN–Fadia and the validity of the results against external data, as previously reported (Tan *et al*, 2006). For the present study, age-specific breast cancer incidence rates for the years 1975–1988, that is, before the implementation of the National Health Service Breast Screening Programme (NHSBSP) were used to estimate age-specific parameters for disease onset. The age-specific breast cancer incidence rates for the year 1988 as simulated by MISCAN–Fadia were compared with the observed incidence rates for the year 1988 in the United Kingdom.

### UK breast screening frequency trial

**Figure 1:**
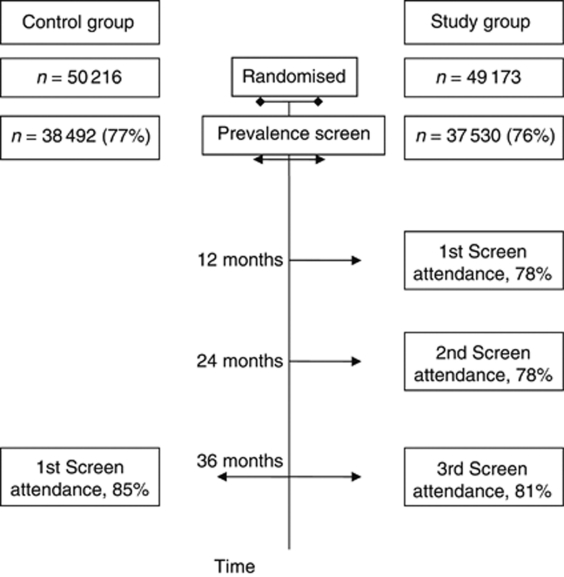
A schematic overview of the UK Breast Screening Frequency Trial.

Five screening units participated in the trial held between 1989 and 1996 (Breast Screening Frequency Trial Group, 2002) (see Figure 1 for an overview of the trial design). A total of 99 389 women aged 50–62 years who had been invited to a prevalence screen in the NHSBSP were randomised to a conventional screen after an interval of 3 years (control group, *n*=50 216), or to three annual screenings (study group, *n*=49 173). For the primary analysis, only women who attended the prevalence screen and in whom no cancer was found at the prevalence screen were included (*n*=38 492 in the control group and *n*=37 530 in the study group). The attendance rate in the control group, among women who had attended the prevalence screen, was 85%. In the study group, attendance rates at the three yearly screens were 78%, 78% and 81%, respectively, (Breast Screening Frequency Trial Group, 2002).

### Trial replication and mortality prediction

Initially, the model based on data from randomised screening trials (extrapolated to the current period) and US data simulated a more favourable tumour size distribution than observed in the trial for both groups. Therefore, the threshold diameter and diameter of clinical detection were estimated using data from the Frequency Trial on the numbers of invasive breast cancers in both groups of the trial split out by tumour size and detection mode (see Appendix). Compared with the initially used values, the estimated values were somewhat higher for the diameter of clinical detection and the threshold diameter, corresponding to a lower screening sensitivity.

Subsequently, this fitted model was used to predict the number of breast cancer deaths from cancers diagnosed during the trial period in each group with a follow-up period up to 2006. From these numbers, a predicted RR of dying from breast cancer in the study group compared with the control group was calculated.

In addition, we investigated the effect of a longer follow-up period (i.e., until all women have died), a higher sensitivity (using the initial value for the threshold diameter) and full compliance (i.e., 100% attendance rates) on the predicted RR.

## Results

### Model calibration and validation

**Figure 2:**
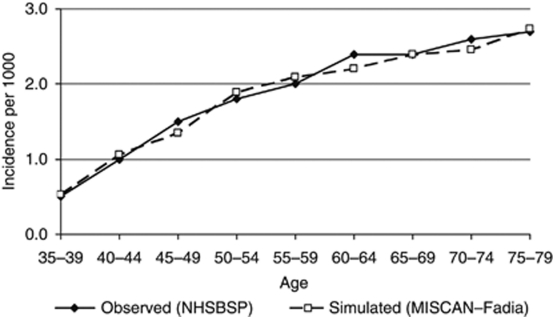
Age-specific breast cancer incidence rates in the UK for the year 1988 as observed and simulated by MISCAN–Fadia.

The observed age-specific incidence rates in the year 1988 as reported by the NHSBSP were accurately reproduced by MISCAN–Fadia (Figure 2). For each 5-year age group (35–79 years), the difference between the observed and simulated incidence rates was <10%.

### Trial replication and mortality prediction

The model with the threshold diameter and diameter of clinical detection estimated based on the trial data, simulated a total of 523 (445 invasive) breast cancers in women who attended the prevalence screen compared with a total of 535 (443 invasive) cancers observed in the trial. The numbers of detected breast cancers and percentages screen detected, and clinically detected cancers are close to the observed numbers and percentages in both the groups (Table 1).

**Figure 3:**
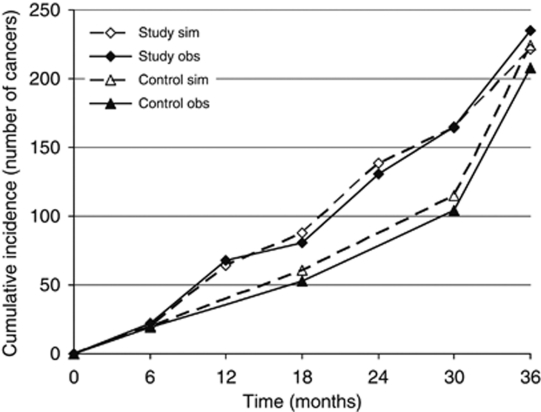
Cumulative incidence (number of invasive breast cancers) over time since prevalence screen in the control and study group as observed in the Frequency Trial (obs) and simulated by MISCAN–Fadia (sim).

For the trial period, the cumulative incidence (number of invasive breast cancers detected) in both groups over time since prevalence screen, as observed in the trial and simulated by the model, is shown in Figure 3.

The model simulated a more favourable tumour size distribution in the study group than in the control group, in line with what was observed in the trial (Table 2). For the control group, the simulated size distribution was somewhat less favourable (61% small tumours simulated *vs* 66% observed) and for the study group, the simulated size distribution was somewhat too favourable (77% small tumours simulated *vs* 73% observed). Thus, the model simulated a larger difference in size distribution between the control and study group than observed.

In the control group, 55 breast cancer deaths from cancers diagnosed in the trial were observed during the median follow-up of 162 months (Duffy and Blamey, 2008), compared with 54 deaths predicted. In the study group 50 breast cancer deaths were observed (Duffy and Blamey, 2008), whereas the model predicted 45 breast cancer deaths. The predicted difference between the number of deaths in the control group and the study group was larger than the observed difference, corresponding to a predicted RR of 0.83 of dying from breast cancer in the study group compared with the control group.

A longer follow-up period (life-time) had no effect on the predicted RR (Table 3). Increasing the sensitivity led to a higher percentage of screen-detected cancers in both groups (78% in the study and 58% in the control group) and to a lower predicted RR of 0.81, as did increasing the attendance to 100%. The combination of full compliance and higher sensitivity led to a predicted RR of 0.77.

## Discussion

The present study suggests that there is benefit in terms of a reduction in breast cancer mortality associated with shortening the screening interval from 3 years to 1 year. The results show that if the available information from the UK Breast Screening Frequency Trial is used in a microsimulation model that is based on the results of randomised screening trials including a large(r) number of women, a larger effect of shortening the screening interval is predicted. The predicted RR of breast cancer death for the study group (offered three annual screens) compared with the control group (offered one screen after 3 years) was 0.83. The effect of more frequent screening is predicted to be larger when the attendance rate or screening test sensitivity is increased.

The microsimulation model used in the present study fitted the data better when a somewhat higher threshold diameter, corresponding to a lower screening test sensitivity, and diameter of clinical detection is used compared with a model based on data from randomised screening trials (extrapolated to the current period) and from the US. Thus, it seems that the screening test sensitivity in the trial was relatively low, which is in line with previously reported results showing that screen-detected as well as interval cancers could benefit from improved sensitivity (Warren *et al*, 2003). The results of the present study indicate that when the screening test sensitivity is higher, the effect of shortening the screening interval will be somewhat larger. This finding is important when considering shorter screening intervals for certain risk groups. For example, it has been hypothesised that women with high breast density might benefit more from additional frequent screening, because they have a higher risk of breast cancer (Kerlikowske *et al*, 2010). However, screening test sensitivity has been found to be lower in women with high breast density (Carney *et al*, 2003). The present study shows that the lower sensitivity in this group might offset some of the potential benefit of more frequent screening in this group.

The most important limitation of the current study is the relative paucity of data available to simulate the trial. More information (e.g., on the age distribution of participants and tumour size distribution of screen *vs* clinically detected cancers in both groups) might further improve the model, and consequently the model predictions. In addition, detailed information on the attendance rates was not available. For example, the non-attendance in the study group was somewhat higher than in the control group (approximately 20% *vs* 15%). It is unknown which proportion of the non-attenders in the study group missed multiple rounds (Andersson, 2002). Including more detailed information in the model will lead to better estimates of the effect of shortening the screening interval.

In addition, only one simulation model was used to estimate the effect of 1-year *vs* 3-year screening intervals. Having multiple models that come to similar findings might have strengthened the conclusion of the present study.

Despite these limitations, the microsimulation model, used in this study, adequately simulated the number of screen detected and interval cancers in both arms of the trial. Moreover, the predicted numbers of breast cancer deaths from cancers diagnosed in the trial were of the same magnitude as that of the reported numbers in both groups (Duffy and Blamey, 2008), and the predicted RR is within the 95% CI of the estimate reported from the trial.

The UK Breast Screening Frequency Trial showed a nonsignificant 7% reduction in breast cancer deaths in the study *vs* the control group (Duffy and Blamey, 2008), whereas the present study finds a substantially larger effect of shortening the screening interval (17%). The question arises why the model outcomes differ from the trial results. Several factors might contribute.

First, the RR predicted by the model is within the 95% CI of the trial-reported RR, indicating that the predicted RR is not statistically different from the trial-reported RR. The Frequency Trial invited 99 389 women, based on an expected 25% difference in breast cancer mortality between the study and control group (Day and Duffy, 1996). The current study shows that this estimated difference of 25% was too optimistic, suggesting that the trial was underpowered to find a significant difference between the two groups. On the basis of the results of the current study (i.e., an RR of 0.83), approximately 945 000 women needed to have been invited for a power of 80% to demonstrate a significant (*P*=0.05) difference in breast cancer mortality between the two groups (Lwanga and Lemeshow, 1991). However, the trial was designed to show a difference in predicted mortality, based on surrogate end points; in this case, the tumour size of the detected cancers. It was estimated that the sample size can be 2.74 times smaller without losing precision when surrogate end points are used (Day and Duffy, 1996). This means that when surrogate end points (such as prognostic indices) are used, at least 345 000 women needed to have been invited in order to have 80% power. Our findings indicate that the number needed to invite can also be reduced by increasing compliance to screening tests or increasing screening test sensitivity.

Furthermore, in the trial, more invasive breast cancers were detected in the study group than in the control group. Thus, more diagnoses have been moved forward in time in the study group than in the control group and then, more breast cancer deaths from cancers diagnosed in trial can be expected in the study group. An alternative would be to compare the number of breast cancer deaths from all breast cancers (during a certain follow-up period). However, after the trial, everyone receives usual care (triennial screening), resulting in a dilution of the effect on mortality. Both comparisons will lead to an underestimation of the effect of more frequent screening (i.e., a bias towards an RR of 1).

The results of the available observational studies are somewhat contradictory on the effect of shortening the screening interval. For example, two retrospective studies showed similar prognostic factors for women screened annually *vs* biennially (White *et al*, 2004; Wai *et al*, 2005). However, two other studies found that women who were screened annually had breast tumours that were smaller and less advanced than those who were screened every other year (Field *et al*, 1998; Hunt *et al*, 1999). Furthermore, six independent models showed that there is some benefit when moving from biennial to annual screening, although the benefit diminished (i.e., the benefit of moving from biennial to annual screening is smaller than that of moving from no screening to biennial screening). For example, 68–90% of the benefit is maintained when moving from annual to biennial screening scenarios for women aged 50–69 years (Mandelblatt *et al*, 2009). Thus, the benefit of screening every year is not three times as large as screening carried out once in every 3 years. The associated harms and costs also have to be taken into account when determining the optimal screening frequency and increases more steeply with more frequent screening than the benefits.

In conclusion, the present study suggests that there is benefit in terms of a mortality reduction of shortening the screening interval from 3 years to 1 year. However, the benefit is probably not large enough to start annual screening (Boer *et al*, 1998). At the same time, there seems to be no reason to abolish the 2-year interval currently used in most European screening programmes. For these programmes, benefits in terms of mortality reductions have been shown (Otto *et al*, 2003; Tabar *et al*, 2003).

## Figures and Tables

**Figure A1:**
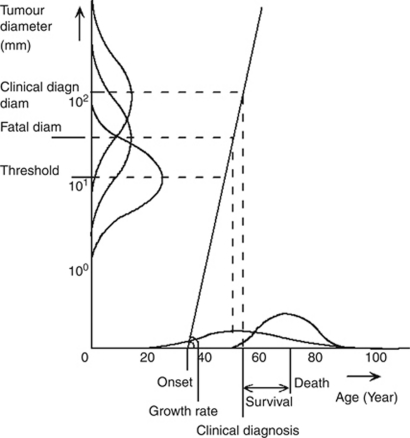
The MISCAN-Fadia natural history model. The model is illustrated by a woman who is diagnosed with incurable breast cancer and for whom screening could have been beneficial. The natural history of breast cancer is simulated through the random selection of six variables from probability distributions, denoted by the various curves: onset=age at tumour onset, growth rate=tumour growth rate, survival=duration between the moment at which the tumour reaches the fatal diameter and the moment of death from breast cancer (not shown), clinical diagn diam=tumour diameter at which the tumour will be diagnosed clinically because of the primary tumour, fatal diam=tumour diameter at which available treatment options will no longer result in cure, threshold diam=tumour diameter at which the tumour becomes screen detectable. After onset the tumour starts growing exponentially according to the tumour growth rate. The diagnosis results from the clinical diagnosis diameter combined with the tumour growth rate. If the tumour is diagnosed after it has reached the fatal diameter, the woman will die from breast cancer. Survival is modelled since fatal diameter. For observed survival (shown), the time between clinical diagnosis and the moment the tumour has reached its fatal diameter has to be subtracted. Screening can change this natural history: After the tumour has reached the threshold diameter, the tumour can be screen detected. If the tumour has not reached the fatal diameter yet at the moment of screen detection, the woman will be cured. Otherwise, screening will not affect the woman's age of death. Reprinted from Tan *et al*, 2006 with permission from Oxford University Press.

**Table 1:** Cumulative number of breast cancers by detection mode in the control group (3-year screening interval) and screen group (1-year screening interval) as observed in the Frequency Trial and simulated by MISCAN–Fadia (prevalence screen attenders only)

	**Observed**				**Simulated**			
	**Control**		**Study**		**Control**		**Study**	
	**Number**	**(%)**	**Number**	**(%)**	**Number**	**(%)**	**Number**	**(%)**
Screen detected	135	(54)	206	(72)	129	(51)	195	(72)
Clinically detected	113	(46)	81	(28)	123	(49)	77	(28)
Total	248		287		251		272	

**Table 2:** Number (%) of detected cancers (invasive by tumour size) in the control and study group as observed in the Frequency Trial and simulated by MISCAN–Fadia

	**Observed**				**Simulated**			
	**Control**		**Study**		**Control**		**Study**	
	**Number**	**(%)**	**Number**	**(%)**	**Number**	**(%)**	**Number**	**(%)**
DCIS	40	(16)	52	(18)	27	(11)	50	(19)
Invasive cancers	208	(84)	235	(82)	224	(89)	227	(81)
*By tumour size*								
1–20 mm	134	(66)	170	(73)	136	(61)	170	(77)
21–50 mm	64	(32)	59	(25)	78	(35)	48	(22)
50+ mm	5	(2)	4	(2)	10	(5)	3	(2)
Not known	5		2		0		0	
Total	248		287		251		272	

Abbreviation: DCIS=ductal carcinoma *in situ*.

**Table 3:** Predicted relative risks using different assumptions

	**Predicted RR**
Base run	0.83
Life-time follow-up	0.83
Higher sensitivity	0.81
100% attendance	0.81
Higher sensitivity and 100% attendance	0.77

Abbreviation: RR=relative risk.

**Table A1:** Maximum likelihood estimates for MISCAN-Fadia natural history parameters and the data sources used to estimate the parameter distribution.

**Variable**	**Distribution**	**Mean**	**s.d.**	**Data used (ref)**
*(a) Parameter estimates and their distribution*				
Growth rate (1/year)	Lognormal (0.062, 0.87)	1.6	1.7	TCS (Tabar *et al*, 1992; Tan *et al*, 2006)
Fatal diameter (cm)	Weibull (4.0, 0.95)	4.1	4.3	SEER (Tan *et al*, 2006)
Survival duration since fatal diameter (years)	Lognormal (2.4, 1.1)	22	35	TCS (Tabar *et al*, 1992; Tan *et al*, 2006)
Diameter at clinical diagnosis because of primary tumour (cm)	Lognormal (0.88, 0.6)	2.8	1.8	Freq (Breast Screening Frequency Trial Group, 2002)
Screening threshold diameters (cm) Age 50–59	Weibull (1.33, 2.95)	1.2	0.4	Freq (Breast Screening Frequency Trial Group, 2002)
Age 60–65	Weibull (1.05, 2.95)	0.9	0.3	Freq (Breast Screening Frequency Trial Group, 2002)
				
**Variable**	***ρ***			
*(b) Correlation between variables*				
Growth rate – survival	−0.9			TCS (Tabar *et al*, 1992; Tan *et al*, 2006)
Growth rate – clinical diagnosis diameter because of the primary tumour	0.41			TCS (Tabar *et al*, 1992; Tan *et al*, 2006)
Clinical diagnosis diameter because of the primary tumour – survival	−0.43			TCS (Tabar *et al*, 1992; Tan *et al*, 2006)
				
(c) *Time since start of fatal disease at which metastases lead to clinical diagnosis of the tumour (fraction of the total survival time after reaching the fatal diameter)*	0.9			TCS (Tabar *et al*, 1992; Tan *et al*, 2006)

Abbreviations: TCS=two county study; s.d.=standard deviation; SEER=surveillance, epidemiology and end results; Freq, UK breast screening frequency trial.

## APPENDIX

This appendix consists of three parts:

1. a model overview containing a description of MISCAN-Fadia,
2. a description of the model components, and
3. a description of the model quantifications of each model component

### Model overview

MISCAN-Fadia (MIcrosimulation of SCreening ANalysis – Fatal diameter) is a microsimulation model generating independent life histories. It uses the so-called parallel universe approach and first simulates the individual life histories for women in the absence of screening and then assesses how these histories would change as a consequence of a screening program. A certain percentage of the modelled population develops pre-clinical disease. The natural history of breast cancer is modelled as a continuously growing tumour. MISCAN-Fadia includes a sub model for ductal carcinoma *in situ* (DCIS), with three different types of preclinical DCIS: regressive DCIS, DCIS that will be diagnosed clinically, and DCIS that will progress to invasive disease. When a screening program is applied, the pre-clinical tumour may be detected by screening if it is larger than a screen-detection threshold diameter.

### Model components

#### Demographics

The demography part of the model simulates individual life histories without breast cancer to form a population. For each person, a date of birth and a date of death of other causes than breast cancer are simulated. The distribution of births and deaths can be adjusted to represent the population simulated.

#### Incidence

A certain percentage of the modelled population develops preclinical disease. This percentage varies between birth cohorts, while the cohorts have the same age distribution of onset of breast cancer.

#### Natural history

Among women who develop disease, the natural history of breast cancer is modelled as a continuously growing tumour. Each tumour has a size (the fatal diameter, which differs between tumours) at which diagnosis and treatment will no longer result in cure given available treatment options. If the tumour is diagnosed (either on the basis of clinical presentation with symptoms or by screening) and treated before the tumour reaches the fatal diameter, the woman will be cured and will die of non-breast cancer causes (Appendix Figure A1). Variation between tumours is modelled by probability distributions of tumour growth rate, threshold diameter of screen detection, clinical diagnosis diameter, fatal disease diameter, and survival duration since fatal diameter.

#### Screening

When a screening program is applied, the preclinical tumour may be detected by screening. Each simulated tumour has a diameter at which it will be clinically diagnosed and a screen-detection threshold diameter. For the latter, screening test sensitivity is 0% below and 100% above this diameter. The threshold diameter is dependent on the calendar year and age of the woman (decreasing with calendar year and older age). Screening benefits result from detection of more tumours at a non-fatal size. The characteristics of organized screening programs, such as screening ages, screening interval and attendance can be specified, and the type of screening (e.g., ‘organized’ or ‘opportunistic’) can be defined in the model.

#### Treatment

The benefit of adjuvant treatment is modelled as a shift in the fatal diameter for treated women. For each adjuvant treatment a cure proportion is estimated (depending on age). These cure proportions are then translated into corresponding fatal diameters (i.e., a more effective treatment can cure a larger tumour). The dissemination of adjuvant treatment is modelled as the probability of being treated with a certain type of treatment (e.g., chemotherapy, tamoxifen).

### Model quantification

#### Demographics

A female population born between 1930 and 1942 (thus, age 50–62 in 1992) was simulated. We assumed that all cohorts were represented in equal proportions. Each woman is assigned a date of death due to non-breast cancer causes based on the UK female population cohort life tables. These life tables were available from the Human Mortality Database (Human Mortality Database, www.mortality.org). The simulated woman dies because of breast cancer or of other causes, whichever comes first.

#### Incidence

We used age-specific breast cancer incidence rates for the years 1975–1988, i.e. before the implementation of the National Health Service Breast Screening Programme to estimate age-specific onset parameters for the onset of breast cancer.

#### Natural history

All parameters were previously estimated using detailed data from the Two County Study (Tabar *et al*, 1992; Tan *et al*, 2006). Subsequently, the fatal diameter was calibrated to U.S. data concerning 1975 stage distribution and 1975 survival (SEER data) (Tan *et al*, 2006).

For the present study, we re-estimated two parameters: the diameter at clinical diagnosis and the screening threshold diameters. We re-estimated these parameters, because these parameters can vary across countries and over time, whereas other parameters (e.g., the tumour growth rate) are assumed to be more or less universal. To estimate these two parameters we used the following data from the UK Frequency Trial:

- The number of detected invasive cancers over time in both groups (control & study group).
- The total number of invasive cancers by tumour size and detection mode in both groups.

The estimated values of the diameter at clinical diagnosis and the screening threshold diameters are shown in Appendix Table A1.

The estimated values for these parameters are somewhat higher than the values previously found based on data from the Two County Study (Lognormal (0.8, 0.6) and Weibull (1.0, 3.0) for the diameter at clinical diagnosis and the screening threshold diameter, respectively) (Tan *et al*, 2006). If these lower values are used to simulate the Frequency Trial, a more favourable tumour size is simulated than the one that is observed in the trial.

#### Screening

In the present study we simulated the screening in the UK Breast Screening Frequency Trial using previously published attendance rates. The attendance rate in the control group, among women who had attended the prevalence screen, was 85%. In the study group, attendance rates at the three yearly screens were 78, 78, and 81%, respectively (Breast Screening Frequency Trial Group, 2002).

#### Treatment

We used treatment effectiveness data based on meta-analyses of the Early Breast Cancer Trialists’ Collaborative Group (Early Breast Cancer Trialists’ Collaborative Group, 1998a, 1998b). The dissemination of adjuvant treatment (the probability of being treated with a certain type of treatment) was based on data from the US (Mariotto *et al*, 2006).
